# Real-Time Safe Landing Zone Identification Based on Airborne LiDAR

**DOI:** 10.3390/s23073491

**Published:** 2023-03-27

**Authors:** Ali Massoud, Ahmed Fahmy, Umar Iqbal, Sidney Givigi, Aboelmagd Noureldin

**Affiliations:** 1 Department of Electrical and Computer Engineering, Queen’s University, Kingston, ON K7L 3N6, Canada; 2 Engineering Physics and Mathematics Department, Zagazig University, Zagazig 7120001, Ash Sharqia Governorate, Egypt; 3 Electrical and Computer Engineering Department, Mississippi State University, Mississippi State, MS 39762, USA; 4 School of Computing, Queen’s University, Kingston, ON K7L 3N6, Canada; 5 Department of Electrical and Computer Engineering, Royal Military College, Kingston, ON K7K 7B4, Canada

**Keywords:** airborne laser scanning, digital surface model, LiDAR, LiDAR point cloud, real-time LiDAR data processing, slope map

## Abstract

Over the past two decades, there has been a growing demand for generating digital surface models (DSMs) in real-time, particularly for aircraft landing in degraded visual environments. Challenging landing environments can hinder a pilot’s ability to accurately navigate, see the ground, and avoid obstacles that may lead to equipment damage or loss of life. While many accurate and robust filtering algorithms for airborne laser scanning (ALS) data have been developed, they are typically computationally expensive. Moreover, these filtering algorithms require high execution times, making them unsuitable for real-time applications. This research aims to design and implement an efficient algorithm that can be used in real-time on limited-resource embedded processors without the need for a supercomputer. The proposed algorithm effectively identifies the best safe landing zone (SLZ) for an aircraft/helicopter based on processing 3D LiDAR point cloud data collected from a LiDAR mounted on the aircraft/helicopter. The algorithm was successfully implemented in C++ in real-time and validated using professional software for flight simulation. By comparing the results with maps, this research demonstrates the ability of the developed method to assist pilots in identifying the safest landing zone for helicopters.

## 1. Introduction

Airborne laser scanning (ALS) is a technology that combines the global navigation satellite system (GNSS), inertial navigation systems (INS), and laser scanners to obtain highly accurate 3D coordinates (x, y, z) of terrain structures. The system uses a multi-sensor fusion of GNSS and INS to determine the position and attitude of the sensor suite, while narrow laser beams measure the range between the suite and target points. Laser scanning is the preferred method due to its efficiency in all environmental conditions, including day and night, as well as in the presence of shadows, sand, snow, rain, and other degraded vision environments [1,2,3,4].

Within the last few years, the ALS has utilized light detection and ranging radar (LiDAR) has been noticeably improved [5,6,7]. It can scan 3D volumes, which results in the production of a 3D point cloud in a short period of time. Consequently, all kinds of buildings, vegetation, obstacles, and flat grounds can be localized, which aids in various types of applications [5].

This research targets using LiDAR sensors mounted on an aircraft to locate safe landing zones (SLZs) [8,9] for aircraft landing in real-time. This is performed first by generating digital surface models (DSM) from raw LiDAR point clouds, followed by the estimation of the slope map from the DSM, and then by the computation of the roughness map from the slope map. Consequently, the SLZs can be identified for aircraft landings. This paper is organized into nine sections, followed by a bibliography. Section 2 reviews the previous work. Section 3 discusses all the proposed methodologies in this paper. It includes the first stage of the proposed method in Section 3.1, in which the raw LiDAR point cloud is prepared for DSM creation. Section 3.2 describes the method used for slope estimation and the DSM creation. SLZ identification is discussed in Section 3.3. Section 3.4 explains the SLZ filtering stage. Upon finishing the methodologies, Section 4 illustrates the experiment setup. The derived results are demonstrated and analyzed in Section 5. Section 6 draws the conclusion and describes future work.

### 1.1. Motivation

Real-time safe landing zone identification based on airborne LiDAR has significant potential impact and applicability to save lives, reduce risk, and improve operational efficiency. There are multiple applications of real-time safe landing zone identification based on airborne data in emergency situations where time is of the essence, such as search and rescue operations or medical evacuations. In these scenarios, promptly and accurately identifying a safe landing zone can mean the difference between life and death. Another application of the proposed technique can be in the context of autonomous drones, flying taxis, and vehicles for obstacle detection and avoidance. Real-time safe landing zone identification is also important in military applications, which include identifying safe landing zones in hostile environments. The application of real-time safe landing zone identification is particularly valuable in urban areas, where finding a suitable landing zone can be challenging due to the presence of buildings and other obstacles.

Situational awareness is crucial during aircraft landings, especially in unclear visual meteorological conditions such as blowing sand, dust, fog, smoke, and darkness, which currently hamper rotary-wing aviation operations. These situations and conditions can potentially cause the loss of equipment and/or lives. Thus, an efficient LiDAR processing algorithm is presented in this paper, capable of generating DSMs and providing a SLZ for the aircraft landing in real-time.

Many algorithms were developed to generate the DSM, but some did not provide a good representation of the ground [8,10,11]. Others have high-quality representation but depend on complex algorithms such as Delaunay triangulation [12,13]. These complex algorithms are computationally expensive and need many simplification techniques to be realized in real-time [14].

### 1.2. Research Contributions

This research paper presents real-time methodologies to find the SLZs from airborne LiDAR data. These methodologies first preprocess the point cloud data collected by the LiDAR. Secondly, it forms a slope and a roughness map of the scanned terrain. A predefined slope and roughness thresholding are applied to the constructed map to identify the SLZs. Finally, the contours of the identified SLZs are determined. All the methods were implemented and examined through real-world scenarios, showing outstanding performance. Because of their practicality, an industrial partner adopted these methods in their helicopter simulator to automatically perform a safe landing using airborne LiDAR.

## 2. Related Work

### 2.1. Digital Surface Map (DSM)

High accuracy and fully automatic acquisition of generating DSM data are one of the photogrammetry technologies [1]. Recently, DSM obtained by airborne LiDAR collecting 3D terrain data has been widely studied. Most of these studies focus on interpolation methods to provide better DSMs.

Problems with the edges in the DSMs produced by triangle-based linear interpolation were noted by Zinger et al. [10]. They found that surfaces are very rough when using the nearest neighbor interpolation, and the oblique surfaces, such as some roof facets, are shown as discontinuous surfaces.

Behan [15] discovered that the use of a grid with a sampling size that relates to the point density at the acquisition phase created the most precise surfaces. Li et al. [8] generated a high-resolution DSM by interpolating all first LiDAR returns through a Delaunay triangulated irregular network (TIN). Since this research used only the first LiDAR return, it sometimes could not accurately represent the ground. This problem occurs when LiDAR point clouds that are very close to each other (relative to their x-y coordinates) have a totally different z-coordinate.

In this case, when triangles are formed, spikes appear in the form of needle-shaped triangles. This adds some distortion to the DSM and eventually leads to faults. Thus, a spike-free algorithm that considers all returns was introduced by another study [12]. This solves the previously mentioned problem, as the algorithm checks all LiDAR points first before constructing the TIN. If this point leads to a spike, it will be ignored completely to avoid any distortion in the digital elevation model (DEM).

### 2.2. Extracting Slope from DSM

The slope is a fundamental parameter in all the studies related to geoscience generally. It can describe the structure of geographic surfaces, analyze topographic surfaces, and accurately monitor the elevation. Moreover, it predicts environmental phenomena such as soil erosion and sediment deposition. Furthermore, it can be used to study water channel morphology and many other applications.

Warren et al. [16] studied ten methods of computing slope from elevation data. They compared these methods with field measurements of the slope. The methods were compared based on the overall estimation of performance, estimation of accuracy, estimation of precision, the independence of the estimation of errors, and the magnitude of field measured slopes. They showed the advantages and limitations of each method and the evolution of these methods over time by researchers. In addition, Vianello et al. [13] compared different methods to calculate the slope from the elevation data, but only for the application of headwater channel network analysis.

Several other algorithms for estimating the slope in a cell-based methodology depend on neighboring elevations [17]. Fleming and Hoffer [18] suggested utilizing only the nearest four neighbors in the 3 × 3 window to calculate the slope of the center cell. Then, Horn [19] presented an eight-neighbor algorithm by differentiating the set of elevations on opposite sides of the central cell. In his equation, the horizontal and vertical neighbors are twice as important as the farther diagonal neighbors.

Chen et al. [20] proposed a new algorithm named Quad to improve slope accuracy. They improve the eight-neighbor algorithm proposed by Horn by using the 2 × 2 cell neighborhood model instead of the 3 × 3 one so that the center cell’s own elevation becomes the most important factor during the calculation. While the horizontal and vertical cells are twice as important as the corner cell, the center cell becomes twice as large as the corner one. They consider the center cell itself, which was not considered by the Horn eight-neighbor algorithm proposed by Horn.

Nie et al. [21] proposed a new physical model that depends on footprints overlapping to estimate terrain slope. This model is used to understand the elevation changes of glaciers in a better way. Their research aims to separate the convolved broadening due to within-footprint slope and roughness and to fully consider the influence of altitude angle, footprint size, shape, orientation, and terrain aspect in the slope calculation process.

### 2.3. SLZ Identification

Previous research (The Autonomous precision Landing and Hazard detection and Avoidance Technology) “ALHAT” has been conducted regarding SLZ identification; it aims to perform precision landing for various types of landing crafts on the moon while avoiding hazards such as rocks, craters, or uneven surfaces [22]. Several flight tests were conducted to develop technology for precise landings on uneven terrain.

LiDAR points can be affected by the motion of the aircraft. Accurate synchronization of the LiDAR system with the aircraft’s motion is crucial to ensuring the correct assignment of accumulated LiDAR points within a given zone. In the real environment, this synchronization is typically achieved by using a single clock in the front end that controls GPS, the inertial navigation system (INS), and LiDAR returns. By synchronizing these systems, the LiDAR points can be precisely aligned with the aircraft’s motion, resulting in more accurate data acquisition.

The work aims to build a complete control system employing sensors, algorithms, and interfaces that control the spacecraft during landing. This enables landing on any planetary body, such as the moon, even if the surface has never been explored before [22].

## 3. Methodologies

### 3.1. LiDAR Point Cloud Preprocessing

**Figure 1:**
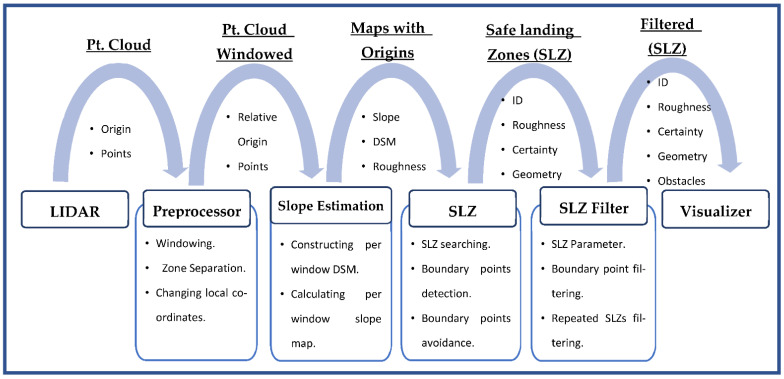
Overall system architecture.

The proposed method suggested in this research deals directly with the LiDAR point cloud to calculate the DSM, slope map, roughness map, and SLZs for aircraft where the LiDAR is mounted. The algorithm consists of four stages: (1) LiDAR point cloud preprocessing. (2) Slope and roughness estimation. (3) Finding SLZ. (4) Filtering SLZ. Figure 1 shows the overall system architecture, including each stage’s inputs and outputs. The preprocessing stage is considered the preparation stage. It prepares the raw LiDAR point cloud for the next stage, in which the slope and roughness will be calculated. Preprocessing is responsible for three tasks: (1) Windowing all the LiDAR point clouds in units of the time window defined by the user. (2) Separating the 3D space into zones, determining the zone in which the aircraft currently exists, and detecting whether the aircraft has moved from one zone to another or not. (3) Changing the point cloud from a local frame relative to the aircraft location to a local frame relative to the zone origin. The preprocessing stage employs different modules to achieve its task. These modules are listed below as follows:

#### 3.1.1. Windowing

The used LiDAR scans the field of view with a high repetition rate, reaching hundreds of scans per second. In each scan, the LiDAR emits laser pulses with a certain pattern and then receives the returned laser pulses due to hitting the objects located in the field of view. By evaluating the time of flight, LiDAR can translate the returned pulses into coordinates for the scanned objects. The coordinates are stored in an array of points, or a so-called point cloud. The number of points in each point cloud is small and insufficient to evaluate the terrain and calculate the slope. Thus, the preprocessing stage aims to accumulate several point clouds within a defined time window. This provides sufficient points to calculate the slope accurately.

#### 3.1.2. Zone Separation

The terrain models (DSM, slope map, and roughness map), which have all the data calculated by the algorithm, have certain dimensions defined by the user. The terrain models should be large enough to cover all the LiDAR point clouds received during the aircraft’s movement for several time windows. The area covered by the terrain models is called the zone. After the movement of the aircraft for many time windows, it will reach the boundary of the zone, meaning that it will receive a point cloud outside this zone.

The important contribution of this module is to separate the 3D space into zones, determine the zone in which the aircraft is currently scanning, and detect if the aircraft has moved from one zone to another. The module detects new zones using the geodetic location of every new scan.

In the real-time implementation, this module first saves the geodetic origin of the first LiDAR point in the first scan. This origin was recorded when the system initially started. Then, the module assumes that a zone is created, with this first LiDAR point as its center. This zone has a rectangular shape with length and width that are determined by the user.

Starting from the second scan, the module always checks if any of the detected points are outside this zone, which means that the aircraft is currently near the zone’s boundaries. Thus, a new zone is created, and the geodetic origin of this LiDAR point is saved and considered its center. In this case, the map creation module in the slope estimation stage creates new terrain models for this zone. The procedure to create new terrain models will be explained in detail later in Section 3.2. Consequently, the same procedure starts again, and the zone separation module repeats itself.

#### 3.1.3. Changing Local Coordinates

This module aims to change the point cloud from a local frame relative to the aircraft location to another local frame relative to the origin of this zone. It saves the geodetic origin of the first LiDAR point in this zone and considers it the origin of the other points in the zone. The Cartesian coordinate of each point is calculated by using Equations (1) and (2).

(1) $$X_{compensated}=X+∆X$$

(2) $$Y_{compensated}=Y+∆Y$$

where:

X: The x-component of the Cartesian coordinate of a LiDAR point.
Y: The y-component of the Cartesian coordinate of a LiDAR point.
∆X and ∆Y are the distances in meters between the position of the aircraft currently, near the boundaries of the zone, and the position of the previous zone origin, in X and Y directions, respectively. They are calculated using Equations (10) and (11), which are explained in Section 3.2.

### 3.2. Slope Estimation

The slope estimation stage is the cornerstone of the whole method suggested in this paper and is where all the intensive processing is conducted. In this stage, the windowed LiDAR point cloud is received from the preprocessing stage. These points are populated to generate the DSM. Consequently, the DSM is used to estimate the slope map and the roughness map. Finally, all the terrain models, including the DSM, slope map, and roughness map, are sent to the SLZ identification stage.

#### 3.2.1. Constructing DSM

For each time window, the preprocessing stage provides a windowed point cloud (${PC}_{w}$), and the algorithm aims to use the windowed point cloud (${PC}_{w}$) to provide the per-window DSM. To do so, the algorithm must filter the outliers’ points from the windowed point cloud (${PC}_{w}$). Then the algorithm will determine the location of this windowed point cloud (${PC}_{w}$) after filtering. After that, it will construct a per-window DSM, which will be used later to construct the total DSM.

##### Outliers’ Points Removal

The windowed point cloud (${PC}_{w}$) received from the preprocessing is not completely ready to be populated in the DSM, as it contains some outliers’ points. Thus, the algorithm filters the outliers’ points in the Y direction by utilizing the histogram filter. This method is also called statistical outlier removal (SOR) [23]. First, the algorithm divides all the given points into a number of bins that can be adjusted by the user, and then it determines the start and end of each bin and accommodates all points into these bins. After that, the algorithm finds the minimum and maximum values; this limits the accepted points, which were determined according to the threshold value. Finally, it filters points so that all points are lower than the minimum value.

##### Defining the Boundaries of the Provided Point in This Time Window

The location of the per-window DSM is determined by the offset and the range of X and Y, where each is defined as:

- The offset is the number of pixels from the beginning of the terrain model to the first changed pixel in this time window;
- The range is the number of pixels between the offset and the farthest-changing pixel in this time window.

The algorithm also filters the range and the offset to make them suitable for the pre-set values, which are defined by the user according to the range and the view angle of the LiDAR, to prevent causing an error when the range is very large. This happens when the farthest received points are away from the first point by a greater distance than the pre-set maximum range.

##### Populating All LiDAR Point Clouds in the Per-Window DSM

The LiDAR point cloud is populated in the per-window DSM according to their X and Y values, where the location of each point in the LiDAR point cloud is determined. This location must be inside one of the pixels in the per-window DSM. The location is determined according to Equations (3) and (4).

(3) $$X_{Pixel}=\frac{X_{Coordinate}-X_{Offset}}{Resolution}$$

(4) $$Y_{Pixel}=\frac{Y_{Coordinate}-Y_{Offset}}{Resolution}$$

Whenever a point from the LiDAR point cloud is found to be inside a pixel of the per-window DSM, the value of this pixel (P) will be equal to the value of the Z component of the point from the LiDAR point cloud. In the case where a pixel has more than one point, the average of the Z component is shown in Equation (5).

(5) $$P=\frac{\sum_{i=0}^{n}Z\left(i\right)}{n}$$

where:

Z: The Z component of a point inside this pixel.
n: The number of points in this pixel.

For the real-time implementation, this step starts with creating two matrices with a size relevant to the range defined in the previous step and a resolution defined by the user. The resolution can be adjusted according to the frequency of LiDAR, the density of the point cloud generated, the speed of the vehicle arraying the LiDAR, and the application. The first one named elevation matrix ($E_{W}$) is basically the per-window DSM. It contains the sum of the altitudes of all the points in each pixel, while the other one, named the elements count matrix ($E_{WC}$) contains the total number of points in each pixel.

After creating the arrays, a loop is executed for all the point clouds. In each of the iterations, the X-coordinate and Y-coordinate of one of the points are checked and matched to the corresponding pixel of the elevation matrix ($E_{W}$) according to Equations (3) and (4).

After the point is matched with a corresponding pixel, this pixel in the elevation matrix ($E_{W}$) is tested to see whether the matched point is the first point added in this pixel or not. In the case where the matched point is the first point added in this pixel, the altitude of this point will be the value of this pixel, and the element count map will be incremented by one when this point is added.

In case the matched point is not the first point added to this pixel, the altitude of this point will be added to the existing value of this pixel, and the element count map will be incremented by one when this point is added. Finally, each element in the elevation matrix ($E_{W}$) is divided by each element in the elements count matrix ($E_{W}C$) and then placed in the elevation matrix ($E_{W}$). Thus, the elevation matrix ($E_{W}$) has the average of all points seen in a certain pixel and will be considered the per-window DSM.

#### 3.2.2. Calculating Slope and Roughness

The slope is the measure of steepness or the degree of inclination of a feature relative to the horizontal plane. The slope is usually expressed as a percentage, an angle, or a ratio. The average slope of terrain can be calculated from a topographic map using the gradient. The slope is the gradient of elevation, so in our case, the slope map is the gradient of the elevation matrix. The method of calculating the gradient is adopted from Burrough et al. [24]. Since the elevation matrix is a two-dimensional array, the gradient of the elevation array is calculated using Equation (6).

(6) $$\nabla E=\frac{\partial E}{\partial x}i+\frac{\partial E}{\partial y}j$$

The roughness map is calculated by differentiating the slope map, which is considered the second differentiation of the elevation array. It is also calculated by using Equation (6).

In real-time applications, the slope needs to be calculated in an iterative way. In this case, we are not dealing with just two points; we are dealing with an elevation vector or matrix, but the methodology used to calculate the slope will be the same. The difference in elevation between every consecutive element in the elevation vector is the vertical distance, while the distance between these two elements is the horizontal distance. Thus, the gradient vector ($\frac{\partial E}{\partial x}$) will be the ratio of the distance between every two elements in the elevation vector (E) over the horizontal distance between them (H). Since the elevation is a 2D matrix, the gradient will be in two directions, X and Y $\left(\frac{\partial E}{\partial x}and\frac{\partial E}{\partial y}\right)$. The equations used to calculate $\frac{\partial E}{\partial x}$ and $\frac{\partial E}{\partial y}$ are (7) and (8), respectively.

(7) $$\frac{\partial E}{\partial x}(i,j)=\frac{E(i,j+1)-E(i,j-1)}{H_{x}(j+1)-H_{x}(j-1)}$$

(8) $$\frac{\partial E}{\partial y}(i,j)=\frac{E(i+1,j)-E(j-1,j)}{H_{y}(i+1)-H_{y}(i-1)}$$

where:

E(i,j): The elements in raw $\left[i\right]$ and column $\left[j\right]$ of the elevation matrix.
$H_{y}(i)$: The horizontal distance in the Y direction of the element in raw $\left[j\right].$
$H_{x}(j)$: The horizontal distance in the X direction of the element in raw $\left[j\right]$.

The per-window slope map ($S_{W}$) is calculated using $\frac{\partial F}{\partial x}$ and $\frac{\partial F}{\partial y}$, not only $\frac{\partial F}{\partial x}$. Thus, it is calculated by the inverse tangent of the mean root square of $\frac{\partial F}{\partial x}$ and $\frac{\partial F}{\partial y}$. The slope matrix elements are calculated as shown in Equation (9).

(9) $$S_{W}(i,j)=\tan^{-1}\left[\sqrt{{\left(\frac{\partial F}{\partial y}(i,j)\right)}^{2}+{\left(\frac{\partial F}{\partial x}(i,j)\right)}^{2}}\right]$$

The per-window roughness map ($R_{W}$) is also calculated the same way as in Equations (7)–(9), but to obtain the roughness matrix, the gradient of the slope is used instead of the gradient of the elevation. In [11], Delaunay TIN was used to estimate the terrain slope. It provides a high-resolution slope since it interpolates all LiDAR returns.

However, the Delaunay TIN method is a computationally expensive technique, and it does not fit our application. As will be shown later in Section 5. Results: A dual-resolution gradient technique based on the number of return points is used to give an asymptotic slope map to the one provided by the Delaunay TIN method.

#### 3.2.3. Constructing the Total Slope Map, the Total DSM, and the Total Roughness Map

After calculating all the per-window terrain models (DSM, slope map, and roughness map), the algorithm begins populating the total path terrain models (DSM, slope map, and roughness map). The algorithm uses the offsets and ranges in X and Y directions to know the position of the per-window map relative to the total map. Then it starts populating the pixels of the per-window map with their corresponding pixels in the total map. The population only occurs if the value of the per-window slope-map pixel is less than the maximum slope; it is of no benefit if the value of the per-window slope-map pixel is higher than the maximum slope. The slope map already has pixels of the maximum slope by default from the initialization.

If the above condition is fulfilled, then the population of elements should occur. The population may occur in two ways, according to the value of this pixel in the slope map.

In case this pixel of the total path slope map was previously empty, the pixel of the total path slope map will have the same value as that of the per-window slope map, the pixel of the total path DSM will have the same value as the per-window DSM, and the pixel of the total path roughness map will have the same value as the per-window roughness map.

If the slope was previously calculated at this pixel, there will be two options in this case:

- The value of that pixel of the total path slope map will be the average of the old one and the new one of the per-window slope maps;
- The value of that pixel of the total path slope map will be the maximum of the two values (the new and the old ones).

In addition, the DSM and the roughness map will have two options for the updating process. These options can be set by the user according to the application. The first option (averaging the two values) is more unbiased because it calculates the average of all readings for the same cell, while the second option (the maximum of the two values) is more cautious because it gives more weight to the higher elevation.

This feature of using the old values of the slope map enables the algorithm to provide better results if the LiDAR is moving at a low speed compared to its frequency, which will enable the algorithm to have more points in order to judge the terrain.

#### 3.2.4. Constructing the Total Binary Map

A total binary map ($B_{T}$) is also constructed to state whether each pixel can be a part of a SLZ or not. A pixel is considered part of a SLZ if the value of the slope in this pixel is above the threshold value. The threshold value is determined by the user according to a safe slope for this special kind of aircraft to land. The values of the pixels of the binary map are calculated in real-time. Where each pixel of the slope map is checked, if the stored value in the slope-map pixel is less than the threshold value, the binary map stores one, indicating that this pixel can be part of an SLZ.

#### 3.2.5. New Map Creation

The movement of the aircraft over long distances will lead to reaching the boundary of the zone, which was created by the zone separation module from the preprocessing stage; this means that it will receive a point cloud outside the zone. The zone separation module will detect this issue and send a flag to the new map creation module. In this case, the responsibility of the new map creation module, is to create new maps for the new zone. It is also responsible for utilizing the pixels of the maps of the previous zone in creating the new map of the new zone.

In creating a new map, the algorithm starts by considering the worst-case scenario, where all the terrain models (DSM, slope map, and roughness map) are filled with the maximum value. So, during the scan, points are gathered and DSM, slope, and roughness for some pixels are calculated, but the rest of the pixels, which did not have any points, will remain at their maximum value. So, they are eliminated from being considered an SLZ.

Then, the module starts by saving all the terrain models of the old zone as temporary terrain models, and it clears the old terrain models. After that, the module starts calculating ∆X and ∆Y, which are the distances in meters between the position of the aircraft currently, near the boundaries of the zone, and the position of the previous zone origin, in X and Y directions, respectively. ∆X and ∆Y are calculated by using Equations (10) and (11) [25].

(10) $$∆X=\left(\emptyset -\emptyset_{o}\right)\left(R+h\right)$$

(11) $$∆Y=\left(\lambda -\lambda_{o}\right)\left(R+h\right)\cos\left(\emptyset \right)$$

where:

∅: The aircraft latitude when reached the zone boundary.
λ: The aircraft longitude when reached the zone boundary.
$\emptyset_{o}$: The latitude of the origin of the previous zone.
$\lambda_{o}$: The longitude of the origin of the previous zone.
R: The radius of Earth.
h: The aircraft altitude when reached the zone boundary.

**Figure 2:**
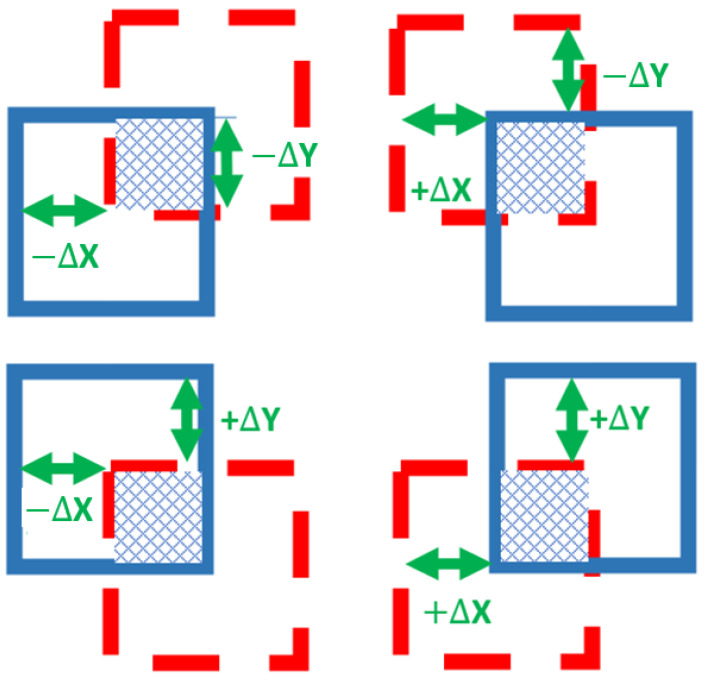
The four cases of intersection area between the old and new zones.

After calculating ∆X and ∆Y, the module begins to benefit from the pixels of the maps of the previous zone, which are also visible in the map of the new zone. In order to do this, the intersection area between the old and the new zones should be determined. The intersection area will be different according to the signs of ∆X and ∆Y, the four cases are shown in Figure 2, where the old zone is marked with a dashed line, the new zone is marked with a solid line, and the intersection area is shaded.

After determining the case according to the signs of ∆X and ∆Y, the values of the shaded area are restored from the temporary terrain models and copied to the new zone.

### 3.3. SLZ Identification

This stage of development is responsible for finding all the SLZs in the binary map array, which was constructed and calculated in the slope estimation stage. Four main tasks are achieved whenever a new windowed terrain model is received from the slope estimation stage. The SLZ identification stage performs these three tasks for each new windowed terrain model it receives.

#### 3.3.1. SLZ Search

This method aims to search for a new SLZ using the total binary map (B_*T*_) constructed in Section 3.2.4, which contains all the safe and unsafe pixels. This method uses the moving window technique, where a moving window is created and moved on every pixel in the binary map in the X and Y directions to check for a safe pixel, as shown in Algorithm 1.

**Algorithm 1:** SLZ Search

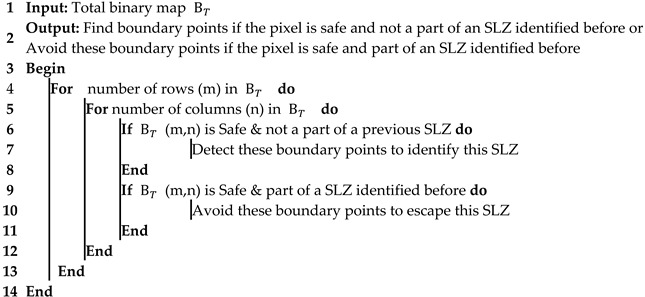



Moreover, the algorithm ensures a margin of unsafe pixels around the map in all directions (up, down, right, and left), because if the algorithm reaches the edge of the map during the search for boundary points, it will not be able to find new neighboring boundary points.

#### 3.3.2. Boundary Point Detection

**Figure 3:**
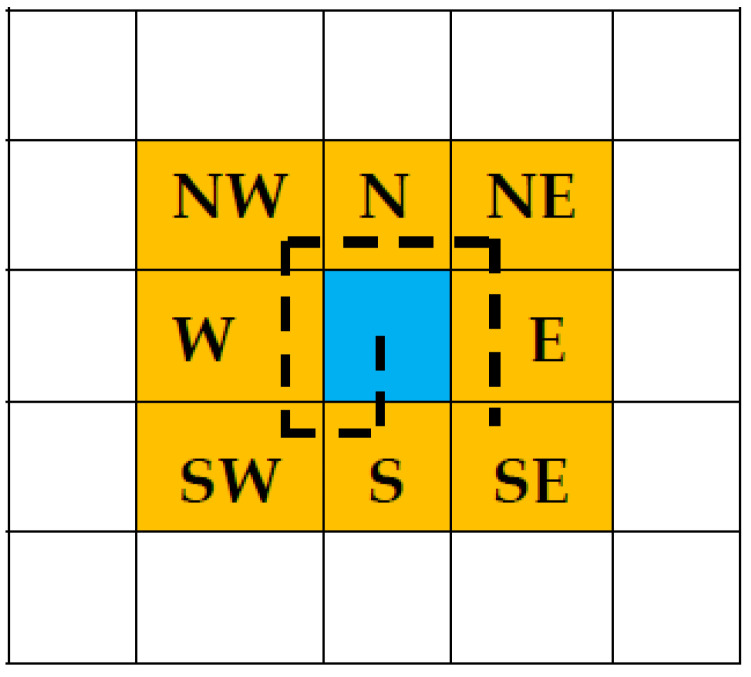
Moore’s Neighbor Pixels. Blue is the pixel of interest, and yellows are the surrounding/neighbor pixels.

The algorithm utilizes the Moore-neighbor algorithm to obtain the next boundary point starting from this pixel [26]. It starts scanning from the top-left position and scans each pixel from left to right downwards. While it traces around the contour in a clockwise direction, it checks the eight neighboring pixels around the first boundary pixel and decides if they are safe, as shown in Figure 3.

When a safe pixel is identified in one of these neighboring pixels, it is considered a new boundary pixel. Consequently, the algorithm starts scanning again, looking for the next neighbor, and so on. The tracing stops when it returns to the initial point or reaches the timeout value.

All the boundary points of this SLZ are saved in addition to the SLZs’ offset and geodetic origin (latitude and longitude). Then the geodetic coordinates (Latitude, Longitude) of the boundary points of this SLZ are calculated and saved using Equations (12) and (13) [25].

(12) $$\emptyset =\left(\emptyset_{o}\right)+\frac{y}{\left(R+z\right)}$$

(13) $$\lambda =\left(\lambda_{o}\right)+\frac{x}{\left(R+z\right)\cos\left(\emptyset \right)}$$

where:

∅: The latitude of the boundary points of this SLZ.
λ: The longitude of the boundary points of this SLZ.
$\emptyset_{o}$: The latitude of the origin of the zone containing this SLZ.
$\lambda_{o}$: The longitude of the origin of the zone containing this SLZ.
y: The y component of the boundary points of this SLZ.
x: The x component of the boundary points of this SLZ.
z: The altitude of the boundary points of this SLZ.
R: The radius of Earth.

Finally, all the boundary points of this SLZ will be marked as confirmed boundary points to prevent counting them again in a new SLZ. For real-time implementation, this method continuously tests all eight Moore-neighbor pixels. Each of the pixels is determined according to Equations (14) and (15).

(14) $$N_{x}=P_{x}+N_{m}(i,0)$$

(15) $$N_{y}=P_{y}+N_{m}(i,1)$$

where:

$N_{x}$: The X component of Moore-neighbor pixel.
$N_{y}$: The Y component of Moore-neighbor pixel.
$P_{x}$: The X component of the previous boundary point.
$P_{y}$: The Y component of the previous boundary point.
$N_{m}$: It is a vector containing the number needed to be added to the previous boundary point to obtain all Moore neighbors. The array is shown in Table 1.
i: The index of the Moore-neighbor vector ($N_{m}$), which ranges from 0 to 8.

**Figure 4:**
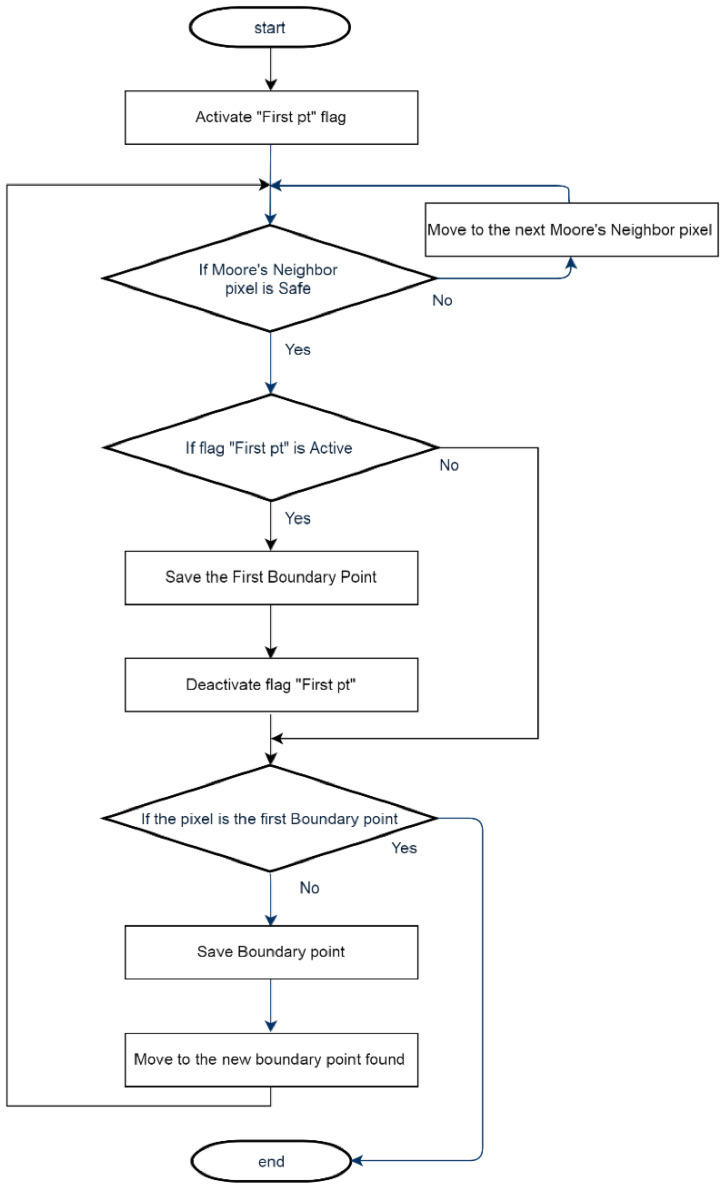
Boundary points’ detection Flowchart.

A flowchart explaining the method is shown in Figure 4.

#### 3.3.3. Boundary Point Avoidance

**Figure 5:**
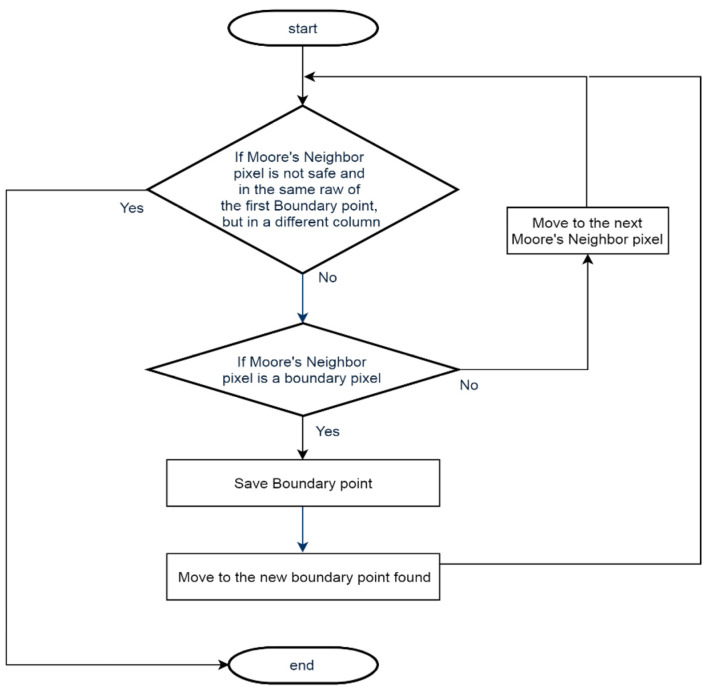
Boundary point avoidance flowchart.

The boundary points avoidance method aims to escape the identified SLZ using the Moore-neighbor algorithm, where it traces the contour until reaching a point that is not a boundary point of any previous SLZ, to be able to start searching again for the safe pixel. The tracing is the same as the one in boundary point detection except for the stopping criteria. The tracing is stopped when it reaches a point in the same row but in another column. This means that the SLZ was successfully avoided, and now the algorithm can search for another SLZ on the map. A flowchart was constructed to emphasize the real-time implementation of the method; the flowchart is shown in Figure 5.

There is another avoidance method for the identified SLZ, where the algorithm moves on the same column while escaping all the safe points. The process takes place until it reaches a point in the same row, but it is not a viable cell, meaning it is not a part of this SLZ.

### 3.4. SLZ Filtering

This stage is responsible for filtering the SLZs to prevent repetition or the intersection of two SLZs. Any SLZ that is considered an update for a previous one uses the same ID to replace the old one instead of drawing two intersected or repeated SLZs. Moreover, it filters the boundary points to exclude collinear points. Additionally, it calculates the certainty and roughness of the SLZ and checks if the SLZ has a square that fits the aircraft’s landing.

The SLZ filtering stage has only one input and one output. Regarding the input, it is all the identified SLZs in the SLZ identification stage. It contains all the needed data for an irregular SLZ, including ID, boundary points, slope, roughness, and content points. Regarding the output, it is also SLZs, but the SLZs after applying all the filters.

This stage has three main tasks that are achieved whenever a new identified SLZ is sent from the SLZ identification stage. This stage completes the tasks and then waits for the next one. These tasks are:

#### 3.4.1. SLZ Parameter Calculation

In this research, we developed a method to find the certainty and roughness of the SLZ and to check if the SLZ has a square-shaped space with a size that is suitable for the landing of the aircraft. Moreover, the same method determines the obstacles inside the SLZ those ones that the aircraft should avoid while landing.

In real-time implementation, the developed method searches for all the SLZ contents cells, which are saved from the SLZ stage. This method finds the number of cells in each of the following groups:

- Safe cells have a slope with a value less than the predetermined slope threshold;
- Unsafe cells have a slope with a value higher than the predetermined slope threshold;
- Uncertain cells, which have no elevation value and mean no LiDAR point, were reflected from this cell;
- Certain unsafe cells, which have an elevation value, have their height determined. In addition to that, they are unsafe cells.

Moreover, the suggested method here accumulates the sum of the roughness of all cells to calculate the total roughness of the cells in the SLZ. In addition, the method calculates the certainty (C) of the current SLZ by using two introduced approaches, with the freedom given to the user to choose any of them according to the application. The two methods are shown in Equations (16) and (17).

(16) $$C_{SLZ}=\frac{P_{S}}{P_{T}}$$

(17) $$C_{SLZ}=\frac{P_{C}}{P_{T}}$$

where:

$C_{SLZ}$: The certainty of this SLZ.
$P_{S}$: The number of safe cells in this SLZ.
$P_{T}$: The total number of cells in this SLZ.
$P_{C}$: The total number of certain cells in this SLZ.

In addition to that, the method saves the location of the unsafe cells inside the SLZ. The location of these cells is converted from Cartesian coordinates to geodetic coordinates by using Equations (12) and (13) [25]. These cells are considered the obstacles inside the SLZ that the aircraft should avoid while landing.

Finally, the method checks if the SLZ has a safe square to land on with the predetermined dimensions set by the user, and it considers for each cell inside the SLZ all the possibilities for discovering a square containing this cell. If a square is found, a flag square is raised to validate this SLZ and prevent checking it again. This saves a lot of computational costs. Otherwise, this SLZ is ignored, as the aircraft cannot be considered safe to land in it.

#### 3.4.2. Boundary Point Filtering

The boundary point filtering method suggested here aims to reduce the number of points that define the SLZ to decrease the size of the structure that contains these boundary points and, consequently, decrease the traffic between the different parts of the system. The process is achieved by excluding the collinear points, whether in the X or Y direction. The real-time implementation of this method is shown in Algorithm 2.

**Algorithm 2:** Boundary Point Filtering

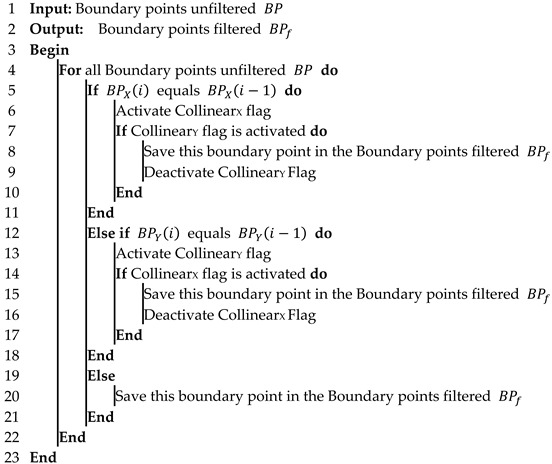



#### 3.4.3. Repeated SLZ Filtering

This filter aims to prevent the repetition or intersection of SLZs, where any SLZ that is considered an update of a previous one takes the same ID of the old one to replace it, instead of drawing two intersected or repeated SLZs. The filter works with the following logic:

- In case the SLZ is completely repeated, this SLZ is ignored;
- In case the SLZ intersects or partially repeats a previous SLZ, the repetition ratio and the area threshold are calculated, and according to their values, the SLZ is considered repeated or not;
- If it is repeated, the new SLZ will replace the previous one;
- If it is not repeated, both the new SLZ and the previous one will remain in effect.

In real-time implementation, this filter checks the repetition of some of the previous SLZs. The number of previous SLZs to be checked is determined by the user. The filter starts by checking the minimum and maximum values of the X and Y components for all the points in the previous SLZs. They are calculated to determine the outer case of the SLZ. The filter also calculates the areas of the previous and current SLZs using the number of content cells inside this SLZ.

Then, the number of boundary points of the current SLZ that are inside the previous SLZ is determined. After that, the repetition ratio (RR) and area threshold ($A_{T}$) are calculated using Equations (18) and (19), respectively:

(18) $$RR=\frac{B_{R}}{B_{T}}$$

(19) $$A_{T}=A_{r}\cdot A_{pSLZ}$$

where:

$B_{R}$: The number of points on the new SLZ that are located inside the previous one.
$B_{T}$: The total number of boundary points of the new SLZ.
$A_{r}$: The minimum ratio between the area of the previous SLZ and that of the current one. (Determined by the user).
$A_{pSLZ}$: Area of the previous SLZ.

The filter uses the repetition ratio (RR) and area threshold ($A_{T}$) to indicate whether these two SLZs are completely repeated, partially repeated, or completely exclusive based on the following logic:

- In cases where the value of the repetition ratio (RR) is one, it is considered the same SLZ, and the new SLZ is ignored;
- In cases where the repetition ratio (RR) is higher than the repetition ratio threshold (determined by the user), and the area of the current SLZ is higher than the area threshold ($A_{T}$), it is considered a repeated SLZ, and the new SLZ will replace the previous one;
- Otherwise, both the new SLZ and the previous one will remain in existence.

It is worth mentioning that the above method identifies safe landing zones (SLZs) before the aircraft comes close to the ground. Thus, LiDAR can clearly detect ground features before the aircraft scatters dust or snow. In cases of heavy snow or heavy rain conditions, the performance will depend on the LiDAR used and the filtering used by the LiDAR manufacturer. Secondly, our algorithm calculates the slope of the terrain and determines the SLZs accordingly. However, it does not guarantee that the surface of the SLZ is strong enough to carry the aircraft’s weight. For example, the SLZ surface may be a weak wooden roof or the surface of a frozen lake, which will not withstand the aircraft weight. Our algorithm does not determine the strength of the SLZ surface. Another challenge our algorithm may also face is when a thick snow blanket might hide the high slope near roof edges.

## 4. Experimental Setup

The proposed algorithm, with all its stages, was fully implemented in C++ and tested to work in real-time. The test was made in a simulation environment on a PC with specifications as shown in Table 2.

The input for the algorithm was a LiDAR point cloud that was obtained in real-time from a LiDAR simulation of the OPAL™ 3D LiDAR scanner from Neptec Technologies with specifications as shown in Table 3.

**Figure 6:**
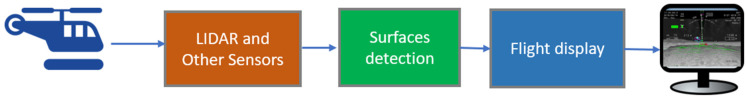
Depiction of Experimental setup.

The SLZ output of the algorithm is displayed using Vega Prime™ software from Presagis, while the DSM, the slope map, the duration of the algorithm, and the number of points are extracted and saved in real-time from the algorithm and plotted after that. A depiction of the experimental setup is shown in Figure 6. The size of the landing zones in this study was defined as 24 m × 24 m, and the size of the scanning process bin was set at 1 m × 1 m. To calculate the number of bins needed to cover the entire area, we divided the total area scanned within the current field of view by the size of each bin. The algorithm uses real-time LiDAR point cloud input obtained from the OPAL™ 3D LiDAR scanner simulation by Neptec Technologies.

The algorithm parameters for all the generated results are shown in Table 4. Vega Prime software displays SLZs for the pilot in real-time. It has two windows for the same scene: the right window is the bird view, and the left window is the pilot view. In addition to that, the Vega Prime software shows SLZs in color code, where SLZs with a dark green color are certain, SLZs with a light green color are less certain, and SLZs with a red color are more uncertain.

## 5. Results and Discussions

### 5.1. SLZ Identification

**Figure 7:**
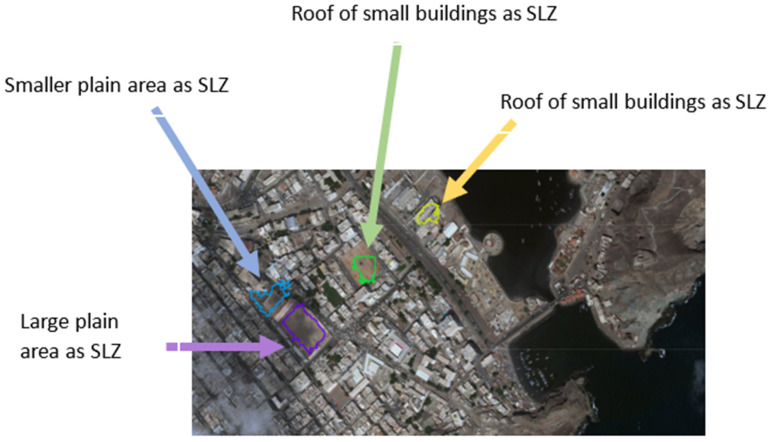
Multiple Safe Landing Zones (SLZ) of Scene 1 projected on an aerial map.

The algorithm’s performance was tested on several scenes in different trajectories to detect SLZs in different conditions, which will face the aircraft during landing. The measurements obtained by each system were converted into Cartesian coordinates to facilitate processing. The resulting data were also represented in Cartesian coordinates, and these findings have been included in the paper. Scene 1, shown in Figure 7, shows SLZs projected on an aerial map; in this map, four SLZs were identified; by analyzing this map after the experiment, it was found that:

- The large plain area could be detected as a safe place to land as it is a grass court in a stadium (violet SLZ); also, the stadium seats are not a part of this SLZ, which shows how accurate it is;
- The smaller plain area could be detected as a safe place to land as it is part of the street and part of the entrance to the stadium (blue SLZ);
- The roof of the large building could be detected as a safe place to land (green SLZ). It is also noticed that some parts of the roof are not included in the SLZ; this is because there was not enough LiDAR point cloud for the rest of the roof;
- The roof of small buildings as a safe place to land (yellow SLZ).

**Figure 8:**
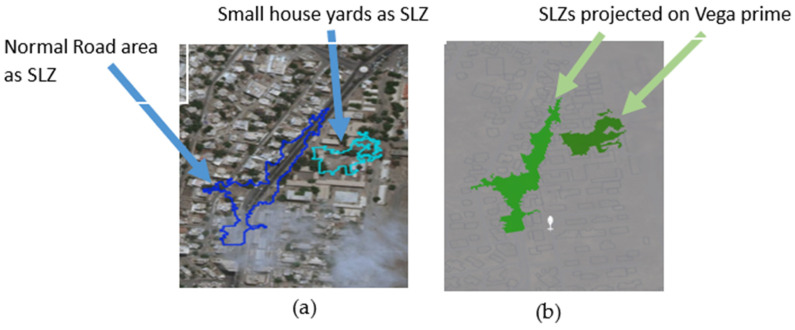
Safe Landing Zones (SLZ) of Scene 2 projected (**a**) on a two-dimensional aerial map and (**b**) on the Vega Prime platform for developing interactive 3D simulations & visualizations.

Moreover, the algorithm was tested in scene 2, the aerial map is shown in Figure 8a, and the map resulting from the Vega Prime software is shown in Figure 8b. In this map, two SLZs were identified, by analyzing this map, it was found that:

- Normal road could be detected as a safe place to land (dark blue SLZ);
- Small house yards could be detected as a safe place to land (light blue SLZ).

**Figure 9:**
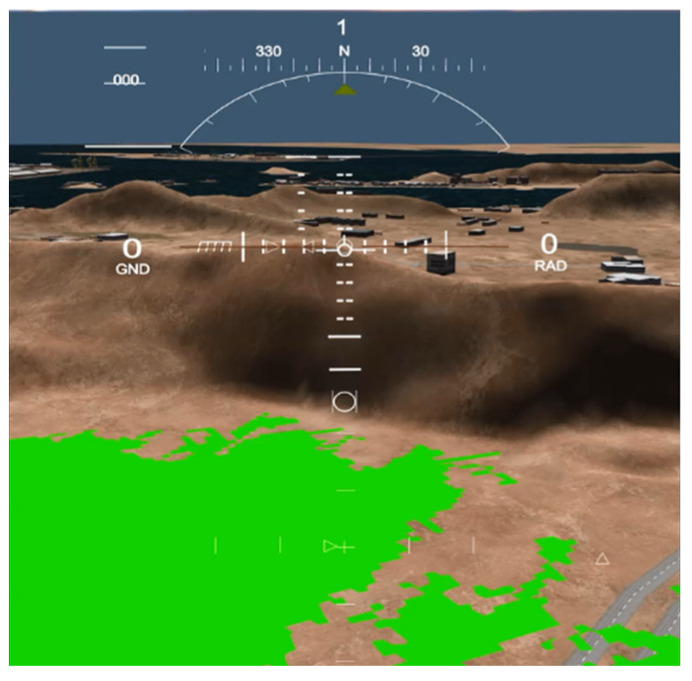
SLZs of Scene 3 projected on Vega Prime.

The algorithm was further tested on the detection of more natural phenomena in scene 3; it was tested for the detection of plain lands between mountains or hills. The algorithm produced a very good result, as shown in Figure 9. It could detect the plain area easily, which was next to a high mountain.

**Figure 10:**
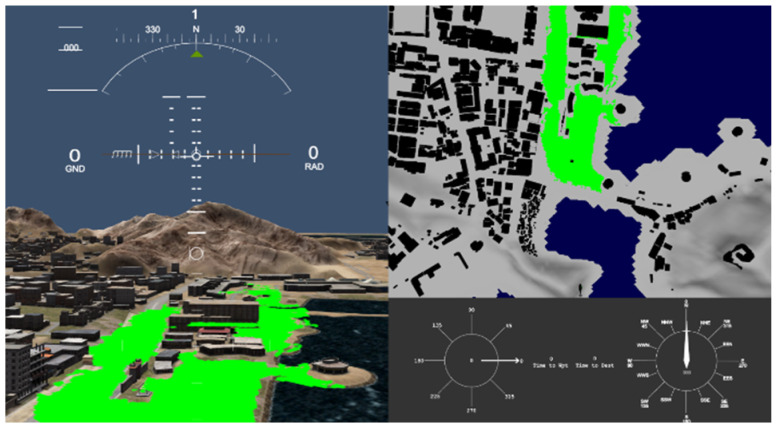
SLZs of Scene 4 projected on Vega Prime.

Finally, the algorithm was tested on a more complicated structure in scene 4. This scene contains many buildings of different heights that are close to each other with small gaps, as shown in Figure 10. The algorithm provides excellent performance by providing all the plain regions, the road, and some of the rooftops as SLZs while avoiding other non-safe regions.

### 5.2. Including Only SLZs Having Enough Space for Landing

**Figure 11:**
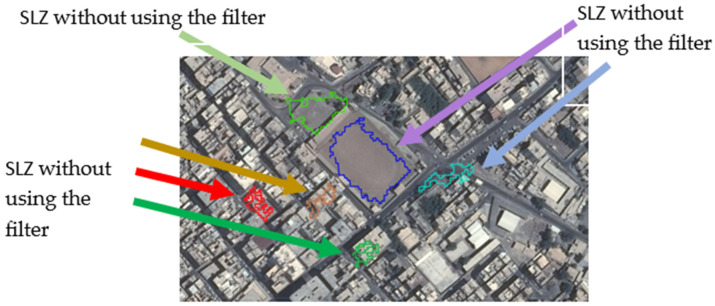
SLZs of parts of Scene 1 projected on the aerial map without using the filter.

**Figure 12:**
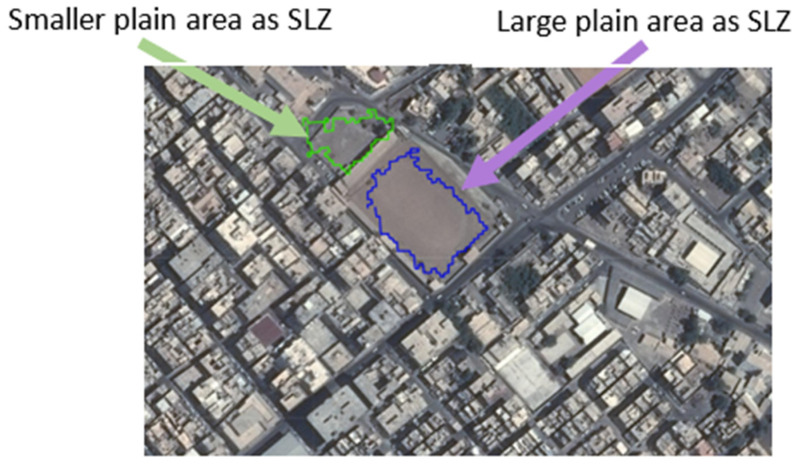
The aerial map has been filtered to identify safe landing zones (SLZs) for a helicopter in Scene 1, and these SLZs are now projected on the map.

The algorithm has a filter that can detect only SLZs that have enough space for the aircraft to land. The filter in this test assumes that the aircraft need a square of 24 m × 24 m to land safely, so all SLZs that do not have this square are excluded. Figure 11 shows part of scene 1 having six SLZ. These SLZs were detected without using the filter. Figure 12 shows the same scene after applying the filter. Four SLZs (red, brown, dark green, and light blue) were excluded. Only two SLZs are detected, which are:

- The court is in a stadium (dark blue SLZ), having a length of 106 m and a width of 73 m;
- The entrance to the stadium (light green SLZ), has a length of 55 m and a width of 62 m.

### 5.3. Re-Use Zone index When It Is a Morphing of an Existing Zone

Another important feature of the algorithm is that it can detect an SLZ that is considered to be morphing onto an existing one. This situation happens a lot, as whenever the aircraft is moving, it will see more points; these points may fill gaps in previous SLZs or add extensions to them. Thus, we need to morph this SLZ into the old one.

**Figure 13:**
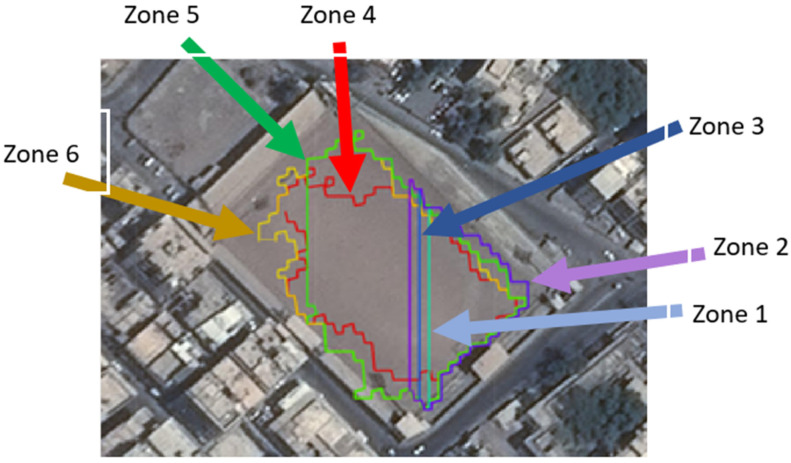
Six safe landing zones (SLZs) have been identified for a helicopter and projected onto an aerial map without using the feature detection method.

**Figure 14:**
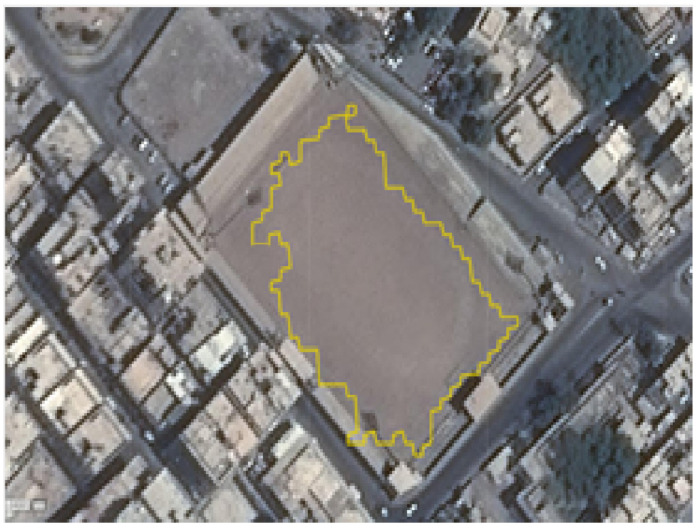
SLZ projected on the aerial map using the feature.

This feature is very important as it prevents an increasing number of SLZs from being identified, which can distract the pilot in addition to overloading the system. Once the algorithm detects that it is a morphing zone, it gives this zone the same index as the old one, so it is considered to be replacing the old one. Figure 13 shows SLZs without using this feature, where six SLZs are detected and shown, while each of them is morphing the previous SLZ. Figure 14 shows only one SLZ after applying the feature.

### 5.4. SLZ Error Evaluation

**Figure 15:**
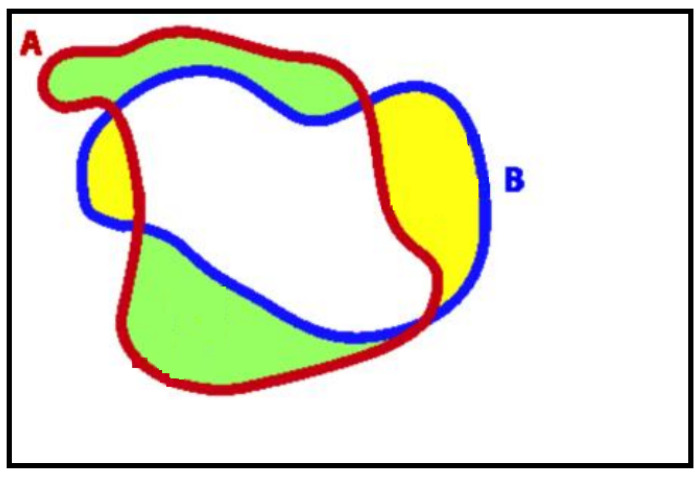
The region-based error between the algorithm’s boundary (A) and the ground truth boundary (B).

A region-based measure is used to evaluate the SLZ error. It determines the error in the regions enclosed by the ground truth contours and the computed boundaries, as depicted in Figure 15.

**Figure 16:**
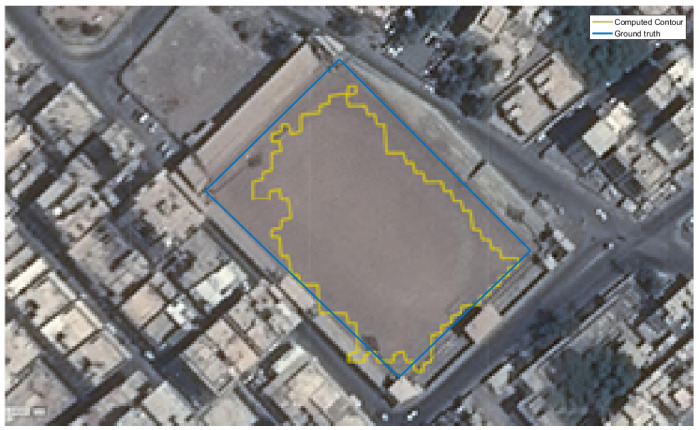
The computed contour versus the ground truth contour.

The error can be expressed as follows [27]:

(20) $$Error=\frac{A_{CT}-A_{GT\cap CT}}{A_{CT}}+\frac{A_{GT}-A_{GT\cap CT}}{A_{GT}}$$

where $A_{CT}$ is the area enclosed by the computed contour, $A_{GT}$ is the area of the ground truth region, and $A_{GT\cap CT}$ is the area of the intersection between the two areas. The first term in (20) penalizes the contour for including unsafe areas in the SLZ. On the other hand, the second term in (20) penalizes the contour for not capturing safe areas defined by the ground truth in the SLZ. The error measure based on this approach ranges between 0 and 2. The minimum value of zero corresponds to identical ground truth and computed contours. Conversely, when the two contours are totally non-overlapping, the error equals 2. In the available data, the safe landing zone is located at the arena, as shown in Figure 16.

In this instance, $A_{CT}$ equals 7078 m^2^, $A_{GT}$ equals 8316 m^2^, and $A_{GT\cap CT}$ equals 6654 m^2^. Given Equation (20), the error is calculated as:

$$Error=\frac{7078-6654}{7078}+\frac{8316-6654}{8316}=0.287$$

The estimated error indicates that we could estimate more than 85% of the ground truth SLZ.

### 5.5. Overall Algorithm Elapsed Time

**Figure 17:**
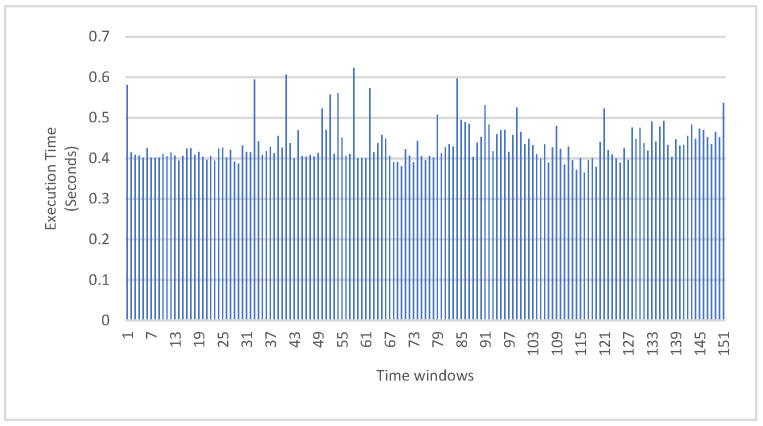
The execution time per time window.

The algorithm was tested in a simulation environment and proved that it could work fully in real-time. The algorithm was executed in less than 0.6 s, for every second of the LiDAR point cloud. The execution time per time window is shown in Figure 17.

## 6. Conclusions and Future Work

Real-time safe landing zone identification based on airborne LiDAR has a significant potential impact. It can save lives, reduce risk, and improve operational efficiency in multiple scenarios, such as search and rescue operations, safe landing zones in hostile environments, and obstacle detection and avoidance in urban areas. The paper presents several techniques to find safe landing zones (SLZs) from airborne LiDAR data in real-time. It introduces a method to extract the slope and roughness map of the scanned terrain with a dual (adaptive) resolution, providing high resolution for areas scanned at low altitudes and a lower resolution for areas scanned at higher altitudes. The method is capable of distinguishing between plain regions, regions with low building density, regions with high building density, and water, even in a degraded visual environment. Based on the extracted map, a predefined threshold is applied to detect potential safe zones for landing. The potential zones with sufficient area for the aircraft are identified as SLZs, and the pilot can select one to land on. The paper also discusses real-world challenging scenarios proposed by an industrial partner where the status of the SLZ may change due to the sudden appearance of the obstacles in the picked zone. The proposed techniques were implemented in C++ and validated by visually comparing the results with the maps, demonstrating their efficiency and suitability for real-time operation.

In future work, the error associated with the estimated SLZs will be quantified, as demonstrated in the SLZ error evaluation section. Moreover, setting parameters such as time window size and the SLZ filter described in Table 4 will be adaptively computed to reduce the error.

## Figures and Tables

**Table 1:** Moore-neighbor vector ($N_{m}$). *i* is the index of the neighbor pixel (0 to 7) total 8. 0 is the relative shift in the x-axis, and 1 is the relative shift in the y-direction.

*i*	0	1
**0**	−1	0
**1**	−1	−1
**2**	0	−1
**3**	1	−1
**4**	1	0
**5**	1	1
**6**	0	1
**7**	−1	1

**Table 2:** PC specifications.

**Operating System**	Windows 7 Enterprise
**Service pack**	1
**RAM**	32.0 GB
**System type**	64 bit
**Processor**	
**Processor count**	2
**Processor Type**	Intel Xeon^®^
**Processor Model**	E5-2650 v4
**# of Cores**	12
**# of Threads**	24
**Processor Base frequency**	2.20 GHZ
**Max Turbo Frequency**	2.90 GHz
**Cache**	30 M.B. (Smart Cache)

**Table 3:** OPAL™ 3D LiDAR.

**Sensor**	
**Range**	Up to 1000 m
**Multi-return**	Up to 7 returns
**Accuracy**	<2.5 cm (typical)
**Precision**	<2.0 cm (typical)
**Laser**	
**Wavelength**	1550 nm
**Physical**	
**Dimensions**	17.8 × 17.8 × 33.8 cm (7.0 × 7.0 × 13.3 inches)
**Weight (without cables)**	11.8 kg (26.0 lbs)
**Operating Voltage**	18–36 VDC
**Power Consumption**	110 W (typical), 220 W maximum

**Table 4:** Algorithm parameters.

Preprocessing Parameters			Comment
**Time window**	1,000,000	The time window (in microseconds)	Each point has a timestamp. The current simulator readout is configured at 1 M every second.
**Slope Estimation Parameters**			
**Max. slope**	40°	The maximum slope	40° is chosen for better visualization of the color map.
**Thre. value**	4°	Slope threshold value	This threshold is defined by the helicopter landing standards.
**SLZ Parameters (irregular)**			
**SLZ Min. Square Side**	24 m	The minimum side length of a landing zone to be considered as an SLZ in the Near mode	This dimension is defined by the helicopter landing standards.
**SLZ Filter**			
**Confidence factor**	0.86	Confidence factor in finding the SLZ certainty	This number is picked high enough to land in a zone that has returns with high power/certainty to avoid false detections.
**Repeat ratio threshold**	0.8	The ratio between the number of boundary points in an old SLZ and that in a new SLZ to be considered as repeated SLZ	As explained, the SLZs with common boundary points and areas combine if these ratios are high enough.
**Area ratio threshold**	0.9	The ratio between the number of area of an old SLZ to that in a new SLZ to be considered as repeated SLZ	

## Data Availability

Our industrial partner provided LiDAR 3D point cloud data for our research project on 4 October 2017 while ensuring compliance with applicable privacy and data protection laws. Presagis|COTS Modeling and Simulation Software.
